# Infarctus rénal bilatéral post-traumatique

**DOI:** 10.11604/pamj.2015.20.376.6761

**Published:** 2015-04-16

**Authors:** Mostafa Rafai, Lahcen Belyamani

**Affiliations:** 1 Service des Urgences Médico-Chirurgicales de l'Hôpital Militaire d'Instruction Mohammed V, CHU Ibn Sina, Rabat, Maroc

**Keywords:** Infarctus, rénal, post-traumatique, Infarctus, renal, post-traumatic

## Image en medicine

L'infarctus rénal post-traumatique est une complication rarement décrite dans la littérature. Jeune patient de 38 ans sans antécédents pathologiques notables, admis au service des urgences pour prise en charge d'un traumatisme grave suite à un accident de la voie publique par choc direct contre le tableau de bord de voiture avec mécanisme de décélération brutale dans le plan horizontal. A l'admission, le patient était stable sur les plans hémodynamique et respiratoire avec un GCS à 14/15. Un body scanner fait après mise en condition initiale objectivait: une dissection de l'aorte descendante, un hémothorax à droite, un épanchement péri-aortique, un pneumopéritoine, une fracture-luxation au niveau D4-D5, une contusion splénique, des zones hypodenses triangulaires rénales bilatérales à base périphérique et à sommet central en rapport avec des infarctus rénaux, et une luxation complète de la tête fémorale gauche associée à une fracture de la colonne postérieure. Le bilan biologique revenait sans anomalies et le patient était admis ensuite au bloc opératoire. L'infarctus rénal post-traumatique peut être secondaire soit à une thrombose soit à une dissection du pédicule artériel rénal. Cependant, parmi les patients victimes d'un traumatisme abdominal, 1 à 4% vont avoir des lésions vasculaires rénales. Ces lésions sont classées en: avulsion, lacération, dissection et occlusion de l'artère rénale. Cette dernière entité pose un problème de prise en charge thérapeutique. Sur le plan physiopathologique, ce type de lésion résulte essentiellement de 2 mécanismes: soit une lésion de l'intima secondaire au traumatisme, soit la compression de l'artère rénale contre la colonne vertébrale.

**Figure 1:**
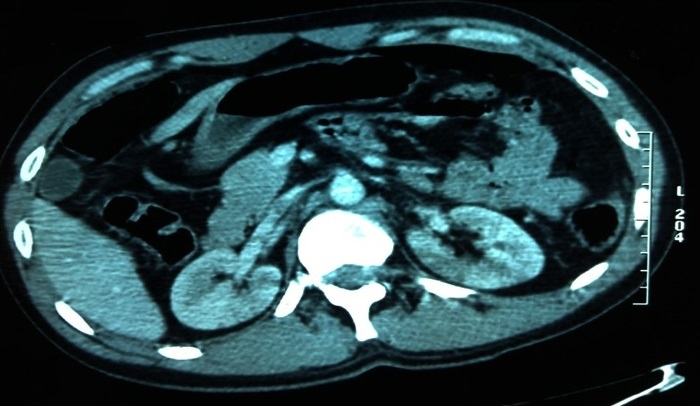
TDM abdominale (coupe transversale): zones hypodenses triangulaires rénales bilatérales à base périphérique et à sommet central en rapport avec des infarctus rénaux

